# Clinical correlates of R1 relaxometry and magnetic susceptibility changes in multiple sclerosis: a multi-parameter quantitative MRI study of brain iron and myelin

**DOI:** 10.1007/s00330-022-09154-y

**Published:** 2022-10-14

**Authors:** Giuseppe Pontillo, Maria Petracca, Serena Monti, Mario Quarantelli, Roberta Lanzillo, Teresa Costabile, Antonio Carotenuto, Fabio Tortora, Andrea Elefante, Vincenzo Brescia Morra, Arturo Brunetti, Giuseppe Palma, Sirio Cocozza

**Affiliations:** 1 Department of Advanced Biomedical Sciences, University “Federico II”, Via Pansini 5, 80131 Naples, Italy; 2 Department of Electrical Engineering and Information Technology, University “Federico II”, Naples, Italy; 3 Department of Neurosciences and Reproductive and Odontostomatological Sciences, University “Federico II”, Naples, Italy; 4 Institute of Biostructure and Bioimaging, National Research Council, Naples, Italy; 5 Multiple Sclerosis Centre, II Division of Neurology, Department of Clinical and Experimental Medicine, “Luigi Vanvitelli” University, Naples, Italy; 6 Institute of Nanotechnology, National Research Council, Lecce, Italy

**Keywords:** Multiple sclerosis, Magnetic resonance imaging, Atrophy, Quantitative susceptibility, Relaxometry

## Abstract

**Objectives:**

The clinical impact of brain microstructural abnormalities in multiple sclerosis (MS) remains elusive. We aimed to characterize the topography of longitudinal relaxation rate (R1) and quantitative susceptibility (χ) changes, as indices of iron and myelin, together with brain atrophy, and to clarify their contribution to cognitive and motor disability in MS.

**Methods:**

In this cross-sectional study, voxel-based morphometry, and voxel-based quantification analyses of R1 and χ maps were conducted in gray matter (GM) and white matter (WM) of 117 MS patients and 53 healthy controls. Voxel-wise between-group differences were assessed with nonparametric permutation tests, while correlations between MRI metrics and clinical variables (global disability, cognitive and motor performance) were assessed both globally and voxel-wise within clusters emerging from the between-group comparisons.

**Results:**

MS patients showed widespread R1 decrease associated with more limited modifications of χ, with atrophy mainly involving deep GM, posterior and infratentorial regions (*p* < 0.02). While R1 and χ showed a parallel reduction in several WM tracts (*p* < 0.001), reduced GM R1 values (*p* < 0.001) were associated with decreased thalamic χ (*p* < 0.001) and small clusters of increased *χ* in the caudate nucleus and prefrontal cortex (*p* < 0.02). In addition to the atrophy, *χ* values in the cingulum and corona radiata correlated with global disability and motor performance, while focal demyelination correlated with cognitive performance (*p* < 0.04).

**Conclusions:**

We confirmed the presence of widespread R1 changes, involving both GM and WM, and atrophy in MS, with less extensive modifications of tissue χ. While atrophy and χ changes are related to global and motor disability, R1 changes are meaningful correlates of cognition.

**Key Points:**

*• Compared to healthy controls, multiple sclerosis patients showed R1 and χ changes suggestive of iron increase within the basal ganglia and reduced iron and myelin content within (subnuclei of) the thalamus.*

*• Thalamic volume and χ changes significantly predicted clinical disability, as well as pulvinar R1 and χ changes, independently from atrophy.*

*• Atrophy-independent R1 and χ changes, suggestive of thalamic iron and myelin depletion, may represent a sensitive marker of subclinical inflammation.*

**Supplementary Information:**

The online version contains supplementary material available at 10.1007/s00330-022-09154-y.

## Introduction

In multiple sclerosis (MS), the physiopathological mechanisms behind atrophy accrual and its impact on disability have been investigated and confirmed by several independent groups [1]. Nevertheless, the nature and clinical relevance of tissue microstructural abnormalities remain more elusive, partly because the coexistence of different pathological processes (demyelination, inflammation, axonal loss) represents a challenge for their characterization [2]. In recent years, semi-quantitative and quantitative MRI (qMR) methods have been developed to explore the nature of microstructural abnormalities, with a particular interest in MS being devoted to the assessment of iron and myelin, as these might offer a glimpse into the neurodegenerative process and tissue repair capability [3, 4]. Indeed, although iron accrual has to be interpreted with caution given the confounding effect of concomitant tissue loss [5, 6], iron depletion in white matter (WM) and in WM-rich structures such as the thalamus likely results from oligodendrocytes dysfunction and damage, with reduced myelination capacity and trophic support leading to neurodegeneration [7]. Among quantitative parameters, histological validation studies have confirmed the applicability of quantitative susceptibility mapping (QSM) for the assessment of iron content in the basal ganglia, where myelin intensities have almost no effect on susceptibility [8]. Within myelin-rich structures such as WM and thalamus, however, interpretation of susceptibility modifications is more challenging, as diamagnetic myelin and paramagnetic iron play opposite effects on susceptibility. In these regions, a susceptibility increase would be an expression of iron accrual/demyelination, while a susceptibility decrease would be anexpression of iron depletion/increase in myelin content.

Another quantitative parameter, which more closely reflects tissue myelin content, is the longitudinal relaxation rate (R1). R1 is strongly associated with both myelin and axon content [9], but, according to postmortem analysis of brain tissue, it is primarily dependent on myelin content [10] and is among the most reliable myelin-sensitive MRI metrics [4]. Beyond the information that susceptibility and R1 changes (as proxies of iron and myelin content) can provide on the nature of microstructural abnormalities in MS, the clinical impact of such modifications remains unclear for several reasons. First, previous studies applying iron and myelin imaging in MS have mainly focused on global disability outcomes [11, 12] or have limited their investigation to selected regions of interest [13, 14]. Second, the fact that correlations with a disability might be driven by atrophy rather than modifications in iron and myelin per se has been rarely investigated [6]. Therefore, the goal of our work was to build on previous findings by exploring the impact of R1 and susceptibility changes on a wide range of disability outcomes (considering also manual dexterity and cognitive function), while accounting for atrophy, which is the main driver of disability accrual in MS [1] and, as recently highlighted, has a major impact on qMR measures [6, 15]. To this aim, we conducted a multi-parametric analysis of quantitative MR, together with brain volumetry, to (i) characterize the topographical distribution of atrophy, R1 and χ changes in the MS brain and (ii) clarify their impact on both cognitive and motor disability.

## Materials and methods

### Subjects

**Fig. 1:**
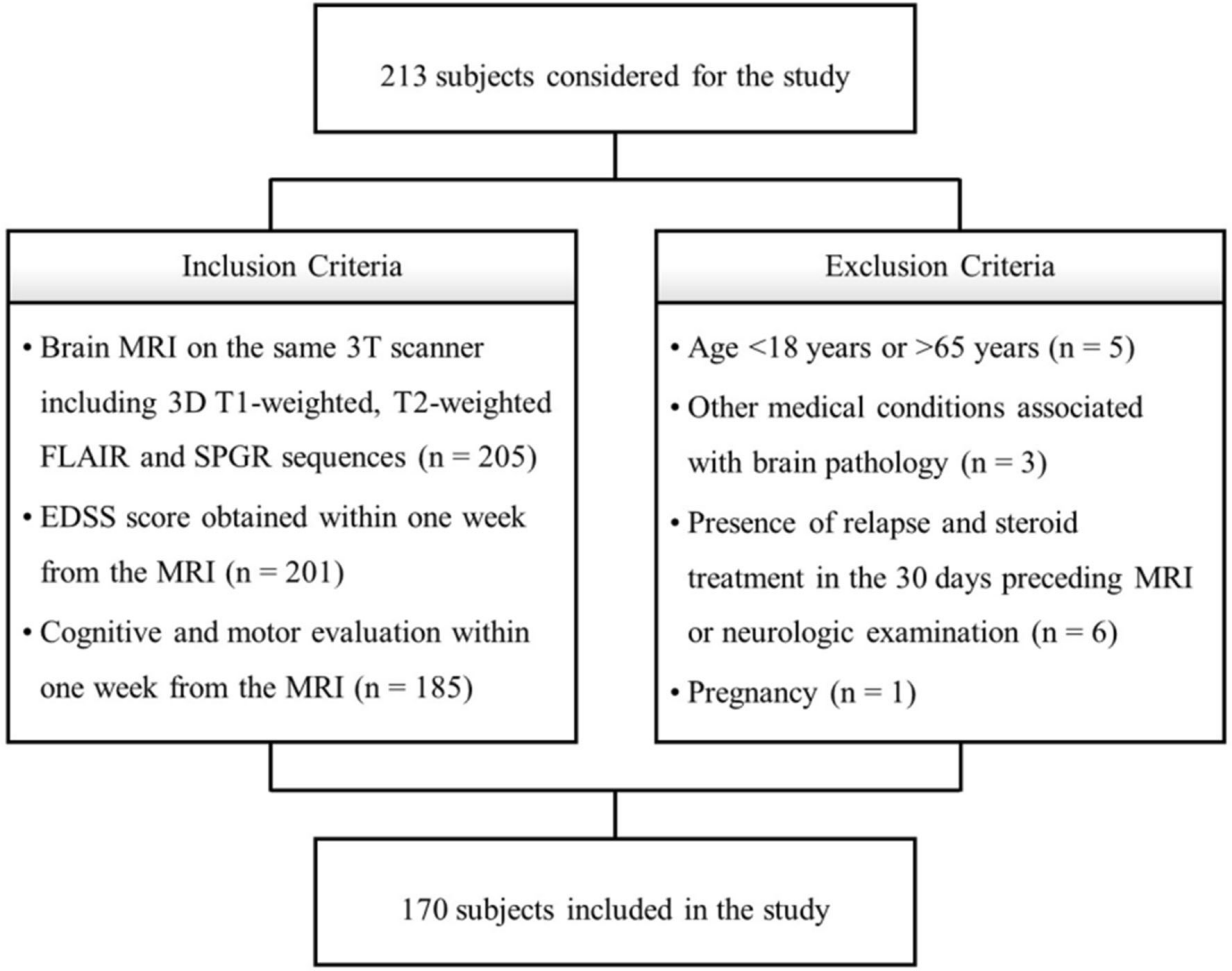
Flowchart showing inclusion and exclusion criteria

In this cross-sectional study, from February 2016 to January 2020, we prospectively enrolled MS patients diagnosed according to the 2010-McDonald criteria [16], along with age- and sex-comparable healthy controls (HC). Inclusion and exclusion criteria are shown in Fig. 1.

The study was conducted in compliance with ethical standards, approved by the local Ethics Committee and written informed consent was obtained from all subjects according to the Declaration of Helsinki.

### Clinical and neuropsychological assessment

Within one week from the MRI, patients’ clinical disability was quantified using the Expanded Disability Status Scale (EDSS) score [17], with disease course classified according to Lublin et al [18]. At the same time, patients were tested using the Symbol Digit Modalities Test (SDMT) [19] to assess cognitive processing speed, while ambulation and manual dexterity were probed through the Timed 25-Foot Walk (T25FW) and the 9-Hole Peg Test (9-HPT) [20], respectively. SDMT scores were expressed as Z-scores with reference to normative values in the healthy population, adjusting for age, gender, and education [19]. Similarly, T25FW and 9-HPT scores were referenced to normative values of an external population of MS patients [20] and averaged to obtain a single composite measure of motor performance. Z-scores were flipped, as appropriate, to have higher scores always corresponding to better performances.

### MRI data acquisition and preprocessing

**Fig. 2:**
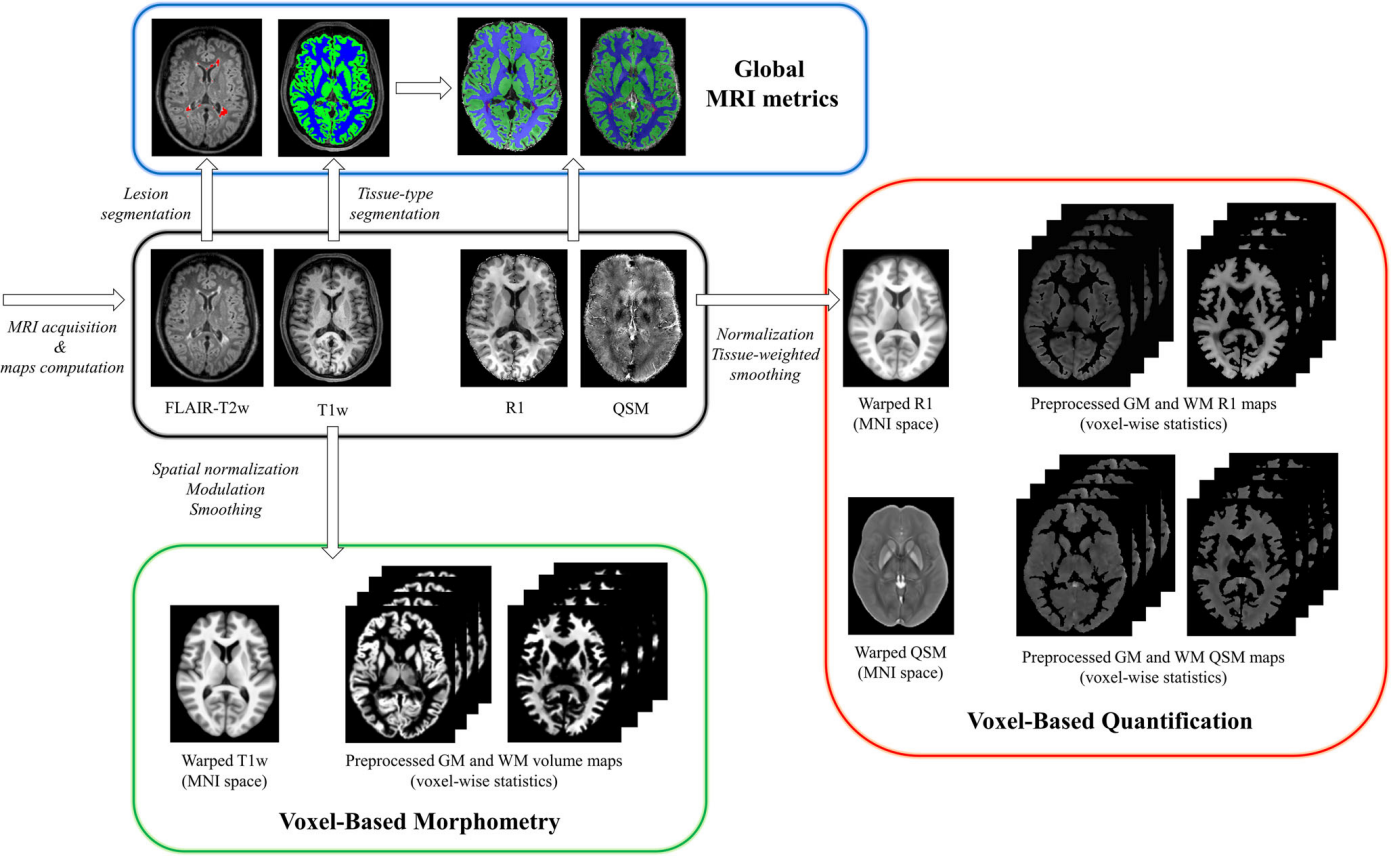
Workflow illustrating the main image processing and analysis steps. Initially, quantitative maps were computed and mapped onto the corresponding T1-weighted volumes and demyelinating lesions were automatically segmented on FLAIR images. For voxel-based analyses, T1-weighted volumes were segmented into different tissue classes and normalized to a 1mm isotropic template in MNI space, with the estimated spatial transformations also applied to quantitative maps. Before entering voxel-wise statistical analyses, normalized gray matter and white matter probability maps were modulated and smoothed, while normalized R1 and χ maps were smoothed via a tissue-weighted smoothing procedure to account for the partial volume contribution of tissue density in each voxel. Using lesion and tissue class masks, global brain volumes and median values of R1 and χ in normal-appearing tissues were also obtained

All MRI exams were performed on the same 3T scanner (Magnetom Trio, Siemens Healthineers) and included a 3D T1-weighted sequence for volumetric analyses, a 3D T2-weighted FLAIR sequence for T2-hyperintense lesions detection, and lesion load (T2-LL) quantification and two spoiled gradient echo sequences for quantitative analyses [21]. Details about acquisition parameters and a thorough description of image preprocessing [22–24], including the computation of R1 and QSM maps [21, 25], are provided in the Supplementary Material, while a flowchart summarizing the main image processing and analysis steps is depicted in Fig. 2.

Briefly, quantitative maps were mapped onto the corresponding T1-weighted volumes, and demyelinating lesions were automatically segmented on FLAIR images via the Lesion Segmentation Tool (LST) toolbox (www.statistical-modelling.de/lst.html) and individual lesion probability maps were used to fill lesions in T1-weighted images and binarized to compute T2-LL.

Subsequent processing steps were carried out following the voxel-based morphometry (VBM) [26] and voxel-based quantification (VBQ) [27] approaches: filled T1-weighted volumes were segmented into different tissue classes and normalized to a 1-mm isotropic template in MNI space via the standard pipeline implemented in the Computational Anatomy Toolbox (CAT12, http://<www.neuro.uni-jena.de/cat), with the estimated spatial transformations also applied to quantitative maps. Finally, normalized GM and WM probability maps were modulated and smoothed using a 1mm full width at half maximum (FWHM) isotropic Gaussian kernel [28]. Instead, normalized R1 and *χ* maps were smoothed (1-mm FWHM isotropic Gaussian kernel) via the tissue-weighted smoothing procedure [27] implemented in the hMRI toolbox (https://hmri-group.github.io/hMRI-toolbox) to account for the partial volume contribution of tissue density in each voxel, resulting in tissue-specific smoothed quantitative maps in MNI space.

For each participant, total intracranial volume (TIV) was also estimated, and brain parenchymal, GM, and WM fractions (BPf, GMf, WMf) were computed. Additionally, individual normal-appearing GM and WM masks were obtained and used to extract median values of R1 and *χ*, while study-specific GM and WM masks were generated to restrict voxel-wise statistical comparisons in order to reduce possible spurious atrophy-related effects on VBQ analyses, as well as to ensure that each voxel was analyzed in only one subspace (i.e., GM or WM).

### Statistical analysis

Unless otherwise specified, statistical analyses were carried out using the Statistical Package for Social Science (SPSSv25.0, IBM corp.) with a significance level *α* = 0.05, and the Benjamini-Hochberg procedure was adopted for controlling the false discovery rate (FDR). Before running parametric analyses, assumptions of the linear model were preliminarily verified [29].

Between-group differences were tested with either Student *t* (age), Pearson chi-square (sex), or age- and sex-corrected ANCOVA (tissue volumes and median R1 and *χ* values) tests.

As for the VBM and VBQ analyses, normalized, modulated, and smoothed tissue probability maps, as well as normalized and smoothed R1 and χ maps, were statistically analyzed, separately for GM and WM, to assess voxel-wise between-group differences using a nonparametric approach based on 5000 permutations applied to the general linear model [30] via the Threshold Free Cluster Enhancement (TFCE) toolbox (http://www.neuro.uni-jena.de/tfce). Age, sex, and TIV were included in the model as confounding variables and previously generated explicit GM and WM masks were used, with a cluster extent threshold *k* = 100 voxels and significance level *p* < 0.05 after correction for multiple comparisons by controlling the family-wise error rate.

When significant between-group differences emerged at the voxel-based analyses, relationships between clinical variables (i.e., EDSS, SDMT, and motor scores) and MRI metrics (tissue probability, R1, and χ maps) were assessed voxel-wise via the TFCE toolbox using regression models with individual clinical scores as the dependent variables and explicit masks restricting the analyses to areas of significant between-group differences. Robust partial correlation analyses, using bootstrap with 5000 resamples, were also carried out between clinical variables and global MRI metrics (i.e., T2-LL and tissue volumes and median R1 and *χ* values), correcting for age, sex, and TIV (for volumes only).

## Results

### Subjects

In total, 117 MS patients (85 relapsing-remitting, 22 secondary-progressive, 10 primary-progressive; 40.6 ± 11.9 years; F/M = 85/32) were included in the study, along with 53 HC (41.3 ± 11.6 years; F/M = 33/20) (Fig. 1). The mean disease duration (DD) for MS patients was 12.7 years (SD: 8.3), with a median EDSS score of 3.0 (interquartile range: 2.0–5.25) and a mean T2-LL of 6.2 ml (SD: 10.7). A total of 110 patients (94.0%) were under immunomodulatory treatment (32% with first-line therapies: interferon, glatiramer acetate, dimethyl fumarate, teriflunomide; 62% with second-line therapies: fingolimod, siponimod, natalizumab, alemtuzumab, ocrelizumab, cladribine) at the time of the MRI.

Demographic and clinical characteristics of the studied population, along with MRI-derived brain volumes and median R1 and *χ* values, are reported in Table 1.

**Table 1:** Demographic, clinical, and MRI characteristics of the studied population

	MS (*n* = 117)	HC (*n* = 53)	*p* value*** (MS vs HC)
*Age (y)*	40.6 ± 11.9	41.3 ± 11.6	0.71
Female sex*	85 (72.6%)	33 (62.3)	0.17
DMT (first line/second line/no therapy)*	38/72/7 (32.5/61.5/6.0%)	-	-
Disease Course (RR/SP/PP)*	85/22/10 (72.6/18.8/9.5%)	-	-
*DD (y)*	12.7 ± 8.3	-	-
EDSS**	3.0 (2.0 - 5.25)	-	-
*Cognitive score*	− 0.82 ± 1.16	-	
*Motor score*	− 0.03 ± 0.86	-	
*T2-LL (ml)*	6.2 ± 10.7	-	-
*BPf (%)*	78.3 ± 4.2	81.0 ± 2.9	**< 0.001**
*GMf (%)*	43.9 ± 2.8	45.3 ± 2.4	**< 0.001**
*WMf (%)*	34.4 ± 2.6	35.7 ±2.0	**< 0.001**
*NAGM median R1 (Hz)*	0.66 ± 0.06	0.70 ± 0.06	** 0.002**
*NAWM median R1 (Hz)*	0.99 ± 0.09	1.06 ± 0.09	**< 0.001**
*NAGM median χ (ppb)*	2.84 ± 1.81	2.44 ± 1.67	0.19
*NAWM median χ (ppb)*	− 9.47 ± 2.58	− 7.97 ± 2.31	**< 0.001**

Unless otherwise indicated, data are expressed as mean ± SD. Between-group differences were tested with either Student *t* (age), Pearson Chi-square (sex), or age- and sex-corrected ANCOVA (MRI-derived measures) tests

*Data are the number of subjects, with percentages in parentheses

**Data are medians, with interquartile ranges in parentheses

***Significant between-group differences are reported in bold

*DMT* disease modifying treatment, *RR* relapsing-remitting, *SP* secondary-progressive, *PP* primary-progressive, *EDS*S Expanded Disability Status Scale, *T2-LL* T2 lesion load, *BPf* brain parenchymal fraction, *GMf* gray matter fraction, *WMf* white matter fraction, *NAGM* normal-appearing gray matter, *NAWM* normal-appearing white matter

### Between-group comparisons

Compared to HC, MS patients had lower GM, WM, and whole-brain volume fractions (*p*-values ≤ 0.001 for all), along with lower median NAGM R1 (*p* = 0.002), NAWM R1, and *χ* (*p* ≤ 0.001) values.

**Fig. 3:**
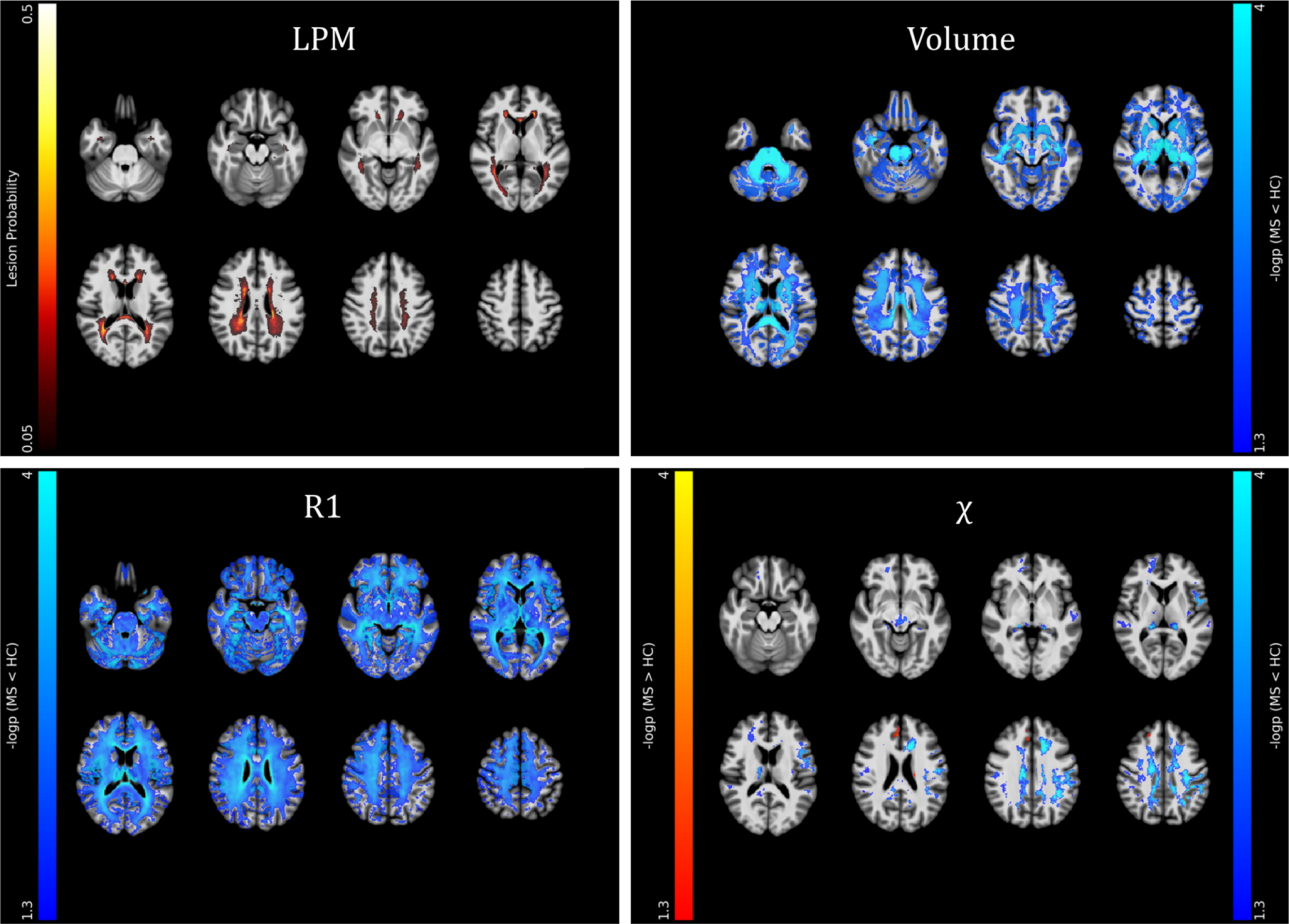
Results of the between-group voxel-wise comparisons. A lesion probability map (LPM), obtained by summing all the binary lesion masks and dividing by the number of patients to give a lesion probability at each voxel, is presented (with a 5% probability threshold, upper left panel), along with clusters of significant between-group difference in terms of volume (upper right panel), R1 and χ (lower panels) values for both the MS > HC (*red-yellow*) and MS < HC (*blue-light blue*) contrasts, all superimposed on axial sections of the average T1-weighted volume in the MNI space. For volume, R1, and χ maps, pooled results of the GM and WM analyses are shown. Images are in radiological orientation

At the voxel-based analyses (Fig. 3 and Supplementary Table 1), MS patients showed massive clusters of reduced volume compared to HC, extensively encompassing both supra- and infra-tentorial GM and WM, with local maxima located in the bilateral thalami and fornices, respectively (*p* values < 0.001). Similarly, widespread clusters of reduced R1 values emerged in MS patients, extending well beyond the distribution of visible T2-hyperintense lesions and peaking in the corpus callosum, periventricular WM, and thalami (*p* values < 0.001). As for the analysis of QSM images, MS patients showed several clusters of reduced *χ* values compared to HC, involving the bilateral cerebral WM (particularly the frontal sections of the corpus callosum, corona radiata, superior longitudinal fasciculus, and cingulum—*p* values < 0.001), the midbrain (*p* = 0.001) and the bilateral pulvinar and right thalamic ventral lateral nucleus (*p*-values < 0.001), along with small clusters of increased χ values in the left body of the caudate nucleus (*p* = 0.004), and the right anterior cingulate (*p* = 0.005) and superior frontal (*p* = 0.02) gyri.

**Fig. 4:**
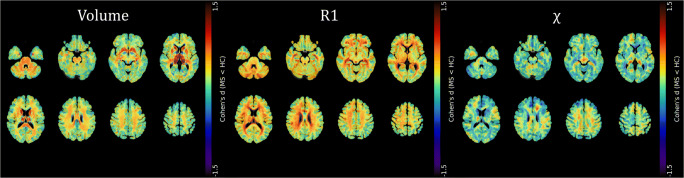
Effect size maps of between-group differences. Effect size (Cohen’s *d*) maps of between-group differences in terms of volume, R1, and χ values (from left to right) are presented, superimposed on axial sections of the average T1-weighted volume in the MNI space. Positive effect size values refer to the MS < HC contrast. For volume, R1, and χ maps, pooled results of the GM and WM analyses are shown. Images are in radiological orientation

Effect size (Cohen’s *d*) maps of between-group differences in terms of regional volume, R1, and *χ* values (obtained from permutation-based T statistics estimated in the TFCE toolbox) are also presented in Fig. 4.

### Relationship between MRI metrics and clinical status

When looking at the relationship with clinical variables, whole brain, and GM volumes were positively associated with motor performance (*r* = 0.245, *p* = 0.009 and *r* = 0.241, *p* = 0.01, respectively), with T2-LL and global GM volume correlating with cognitive processing speed (*r* = − 0.255, *p* = 0.006 and *r* = 0.234, *p* = 0.01, respectively) and more weakly with clinical disability (*r* = 0.200, *p* = 0.03 and *r* = − 0.203, *p* = 0.03, respectively, not surviving multiple comparisons correction). No significant correlations emerged between clinical status and median R1 and χ values of both NAGM and NAWM (Table 2 and Supplementary Figure 2).

**Table 2:** Correlations between clinical and MRI-derived variables. Results are expressed as correlation coefficients (*r*) with 95% bias-corrected and accelerated bootstrap confidence intervals in parentheses (first row) and corresponding *p*-values (second row)

	EDSS	SDMT	Motor score
*Global MRI metrics*^*a*^			
*T2-LL*	0.200 (0.033, 0.407) 0.03*	− 0.255 (− 0.360, − 0.151) **0.006**	− 0.164 (− 0.324, − 0.029) 0.09
*Whole brain volume*	− 0.161 (− 0.356, 0.024) 0.09	0.177 (0.011, 0.339) 0.06	0.245 (0.078, 0.413) **0.009**
*GM volume*	− 0.203 (− 0.370, − 0.027) 0.03*	0.234 (0.075, 0.386) **0.01**	0.241 (0.089, 0.381) **0.01**
*WM volume*	− 0.061 (− 0.246, 0.112) 0.52	0.057 (− 0.158, 0.257) 0.55	0.153 (− 0.016, 0.332) 0.11
*NAGM median R1*	− 0.136 (− 0.313, 0.053) 0.15	0.076 (− 0.094, 0.250) 0.42	0.039 (− 0.132, 0.210) 0.68
*NAWM median R1*	− 0,158 (− 0.331, 0.030) 0.10	0.167 (− 0.009, 0.338) 0.08	0.118 (− 0.042, 0.278) 0.22
*NAGM median χ*	0.007 (− 0.187, 0.196) 0.94	− 0.019 (− 0.201, 0.167) 0.84	− 0.022 (− 0.224, 0.193) 0.82
*NAWM median χ*	− 0.084 (− 0.291, 0.140) 0.38	− 0.030 (− 0.200, 0.141) 0.75	0.136 (− 0.049, 0.303) 0.15

Significant results are in bold

^a^Correlations with clinical scores are corrected for age, sex, and TIV (for volumes only)

*Not significant after FDR correction

*EDSS* Expanded Disability Status Scale, *SDMT* Symbol Digit Modalities Test, *T2-LL* T2 lesion load, *GM* ray matter, *WM* = white matter, *NAGM* normal-appearing gray matter, *NAWM* normal-appearing white matter

**Fig. 5:**
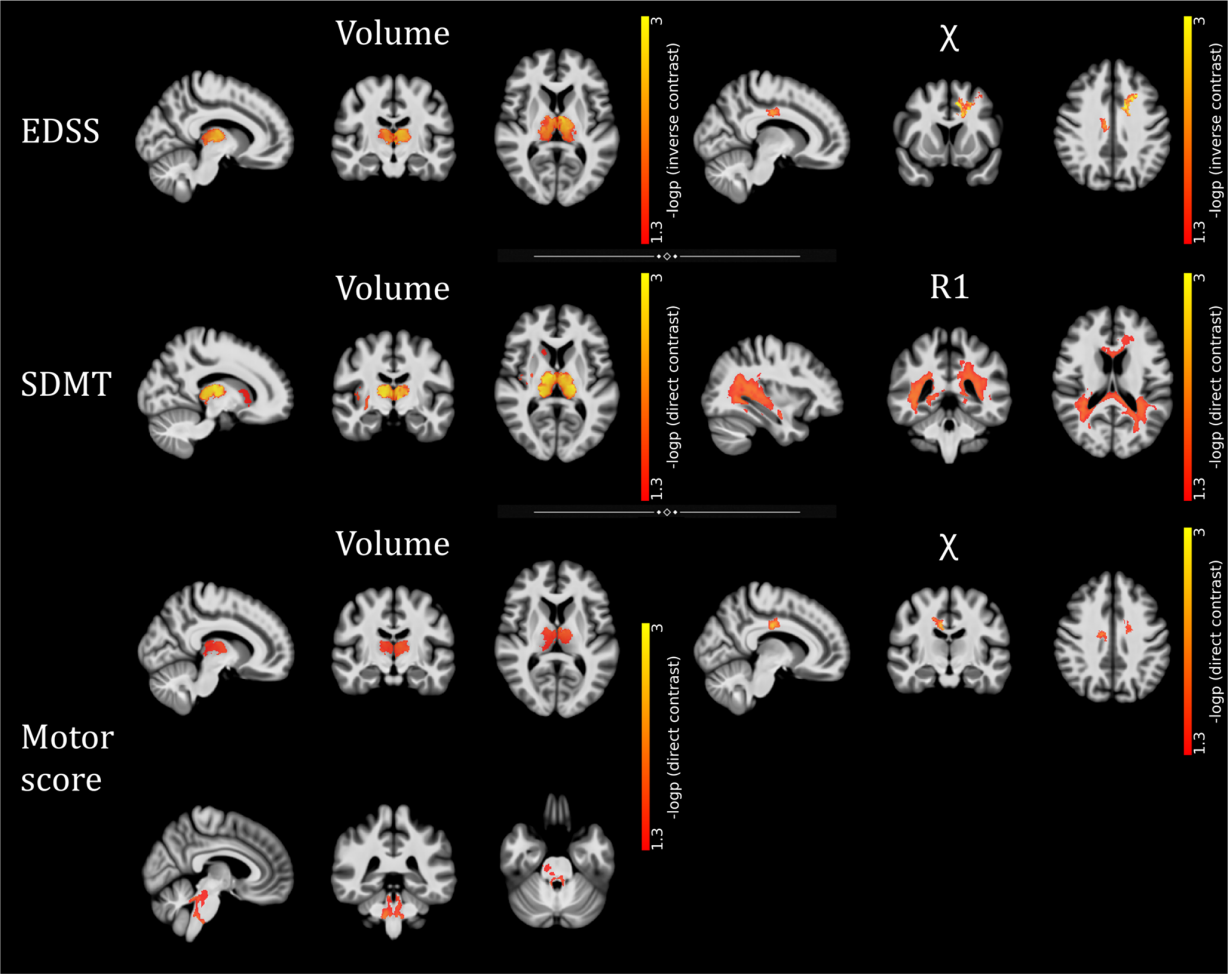
Results of the voxel-wise correlations with clinical variables. Clusters of significant association between MRI metrics and EDSS, SDMT, and motor (from top to bottom) scores are presented, superimposed on sagittal, coronal, and axial (from left to right) sections of the average T1-weighted volume in the MNI space. Images are in radiological orientation

To allow for precise anatomical localization of the effects of interest, associations with clinical variables were also tested at the voxel level (Fig. 5 and Supplementary Table 2): thalamic volume was related both negatively with clinical disability (*p* = 0.001) and positively with cognitive processing speed (*p* = 0.001) and motor performance (*p* = 0.01), with additional positive correlations between the SDMT score and GM volume in the right basal ganglia and posterior insula (*p* values < 0.04) and between motor performance and infratentorial WM volume at the level of the medial lemnisci and cerebellar peduncles (*p* = 0.01). Furthermore, a large cluster of significant association between the SDMT score and R1 values emerged, extensively involving the (mainly posterior) periventricular WM and peaking around the right posterior thalamic radiation (*p* reaching 0.01), while *χ* values in the frontal sections of the cingulum and corona radiata were related both negatively with EDSS (*p* reaching < 0.001) and positively with motor performance (*p* reaching 0.003).

## Discussion

Notwithstanding the many advances witnessed in the field of tissue microstructure in MS, none of the available MR techniques is solely affected by a specific pathological aspect, which advocates for the application of multi-parameter approaches to improve our understanding of microstructural damage [25, 31]. Here, we applied a multi-parameter analysis of volumetry and quantitative MRI to address the following questions: (i) what is the topography of iron and myelin changes assessed via susceptibility and relaxometry in MS? and (ii) are the observed changes clinically relevant?

As per the first question, MS patients showed a widespread R1 decrease across GM and WM regions, associated with substantially more limited modifications in susceptibility and with an atrophy pattern mainly involving deep GM, posterior and infratentorial regions. The observed R1 reduction throughout the WM was likely driven by changes in macromolecular tissue content (i.e., myelin), and, to a lesser extent, by iron levels [32]. Indeed, pathological descriptions documented demyelination not only in the context of lesions but also in normal-appearing WM [33], with iron depletion in remyelinated plaques [7] also possibly contributing, to a lesser extent, to R1 reduction. The R1 voxel-wise analysis confirms and expands previous findings reporting R1 decrease in multiple WM tracts in MS compared to HC [4], with no specific regional preference [34]. Similarly, the parallel susceptibility decrease in several WM regions confirms histopathological data reporting iron depletion in normal-appearing WM [7] and adds to a recent study reporting a decrease in susceptibility within the cingulum in a relatively small group of patients [35]. Indeed, our larger sample size and the application of a voxel-wise approach likely explain the increased sensitivity to the detection of between-group differences. Nevertheless, given previous histopathological data reporting a significant decrease of iron in oligodendrocytes and myelin within normal-appearing WM [7], the limited spatial extension of the observed susceptibility reduction is somehow surprising. A possible explanation could be that, as myelin and iron exert opposite effects on susceptibility, our ability to investigate tissues characterized by concomitant presence of demyelination and iron depletion remains intrinsically limited.

In GM, the observed R1 reduction was associated with decreased susceptibility in the thalamus and small clusters of increased susceptibility in the caudate nucleus and cortical areas. While R1 in the cortex and thalamus can be considered a reliable marker of myelin content [6, 36], in the basal ganglia it is highly influenced by iron concentration [6]. The observation that the thalamus undergoes structural modifications similar to those observed in WM, with demyelination associated with iron depletion, confirms recent findings [6, 12, 37, 38] and can be explained by its peculiar anatomical structure. Likewise, our findings of small areas of increased susceptibility in the cortex and caudate nucleus are in line with recent reports, suggesting that susceptibility increase in MS deep GM, originally interpreted as demyelination and iron accrual [11, 12, 14], is mainly accounted for by atrophy rather than an actual increase in iron content [5, 6].

As per the clinical meaning of microstructural abnormalities, R1 changes seem to reflect the impact of focal lesions, with the cluster holding a significant correlation with cognitive performance peaking in the periventricular region, in overlap with focal demyelination, likely causing disconnection of distributed networks responsible for the control of high-level functions. On the other hand, susceptibility and atrophy, reflecting oligodendrocyte and axonal damage, significantly contributed to global and motor disability. Our analysis confirmed not only the central role of thalamic atrophy as a meaningful correlate of disability in MS [39] but also the relevance of cerebellar WM damage in driving motor impairment [40]. Beyond these confirmations, we identified a correlation between susceptibility reduction and disability that might add another layer to our understanding of the pathological mechanisms sustaining clinical impairment in MS. Indeed, if susceptibility decrease is the expression of oligodendrocyte damage [7], the consequent reduction of the tissue repair capability, depending upon oligodendrocyte activity, would contribute to the manifestation of clinical deficits. On the other hand, cognitive performance was related to deep GM atrophy and R1 abnormalities mostly overlapping the distribution of focal WM lesions. Such overlap, together with the correlation identified between lesion load and SDMT, suggests that, rather than microstructural damage, GM atrophy and disconnection sustained by focal lesions remain the main predictors of cognitive dysfunction in MS [41].

Our work is not without limitations. First, our assessment was conducted on the entire GM and WM rather than the normal-appearing tissue. However, when comparing extra-lesional median R1/susceptibility values at the group level, we identified significant differences, demonstrating that microstructural abnormalities also affect normal-appearing tissue. Additionally, the voxel-based analysis clearly demonstrated that such modifications are not spatially restricted to areas affected by T2 hyperintense lesions, as R1/susceptibility alterations are not only present where lesion have a higher probability to occur, but are also identified within normal-appearing tissue. Finally, although our clinical evaluation included motor and cognitive assessments, and our approach allowed for a multifaceted exploration of tissue abnormalities, we are still far from a comprehensive characterization of the structural substrate underpinning clinical disability in MS.

In conclusion, we confirmed the presence of widespread and clinically relevant demyelination, expressed by R1 decrease, and atrophy in MS. In addition, our findings suggest that also the more limited modifications of tissue susceptibility are clinically meaningful, possibly adding information on oligodendrocyte dysfunction and damage to the ones provided by demyelination and atrophy estimation.

## Supplementary information

Supplementary Figure 1 (PNG 115 kb)

High Resolution Image (TIFF 309 kb)

Supplementary Figure 2 (PNG 333 kb)

High Resolution Image (TIFF 512 kb)

ESM 2 (DOCX 55.2 kb)

## Abbreviations

DD: Disease duration
EDSS: Expanded disability status scale
GM: Gray matter
HC: Healthy controls
MS: Multiple sclerosis
NAGM: Normal-appearing gray matter
NAWM: Normal-appearing white matter
QSM: Quantitative susceptibility mapping
T2-LL: T2 lesion load
TIV: Total intracranial volume
WM: White matter

## Declarations

### Guarantor

The scientific guarantor of this publication is Giuseppe Pontillo, MD.

### Statistics and biometry

One of the authors has significant statistical expertise.

### Methodology

• prospective

• cross-sectional study

• performed at one institution
