# Correction to: Clinical correlates of R1 relaxometry and magnetic susceptibility changes in multiple sclerosis: a multi-parameter quantitative MRI study of brain iron and myelin

**DOI:** 10.1007/s00330-022-09279-0

**Published:** 2022-12-08

**Authors:** Giuseppe Pontillo, Maria Petracca, Serena Monti, Mario Quarantelli, Roberta Lanzillo, Teresa Costabile, Antonio Carotenuto, Fabio Tortora, Andrea Elefante, Vincenzo Brescia Morra, Arturo Brunetti, Giuseppe Palma, Sirio Cocozza

**Affiliations:** 1 Department of Advanced Biomedical Sciences, University “Federico II”, Via Pansini 5, 80131 Naples, Italy; 2 Department of Electrical Engineering and Information Technology, University “Federico II”, Naples, Italy; 3 Department of Neurosciences and Reproductive and Odontostomatological Sciences, University “Federico II”, Naples, Italy; 4 Institute of Biostructure and Bioimaging, National Research Council, Naples, Italy; 5 Multiple Sclerosis Centre, II Division of Neurology, Department of Clinical and Experimental Medicine, “Luigi Vanvitelli” University, Naples, Italy; 6 Institute of Nanotechnology, National Research Council, Lecce, Italy

**Correction to: European Radiology**

10.1007/s00330-022-09154-y

The original version of this article, published on 14 October 2022, unfortunately contained a mistake. The affiliation of the author Giuseppe Palma was incorrectly given as ‘Institute of Biostructure and Bioimaging, National Research Council, Naples, Italy’ and has now been corrected to ‘Institute of Nanotechnology, National Research Council, Lecce, Italy’. The original article has been corrected.

