# Supplementary figures and images for: Clinical correlates of R1 relaxometry and magnetic susceptibility changes in multiple sclerosis: a multi-parameter quantitative MRI study of brain iron and myelin
Source: Eur Radiol. 2022 Oct 14;33(3):2185–94. doi: 10.1007/s00330-022-09154-y (PMC9935712; doi:10.1007/s00330-022-09154-y)

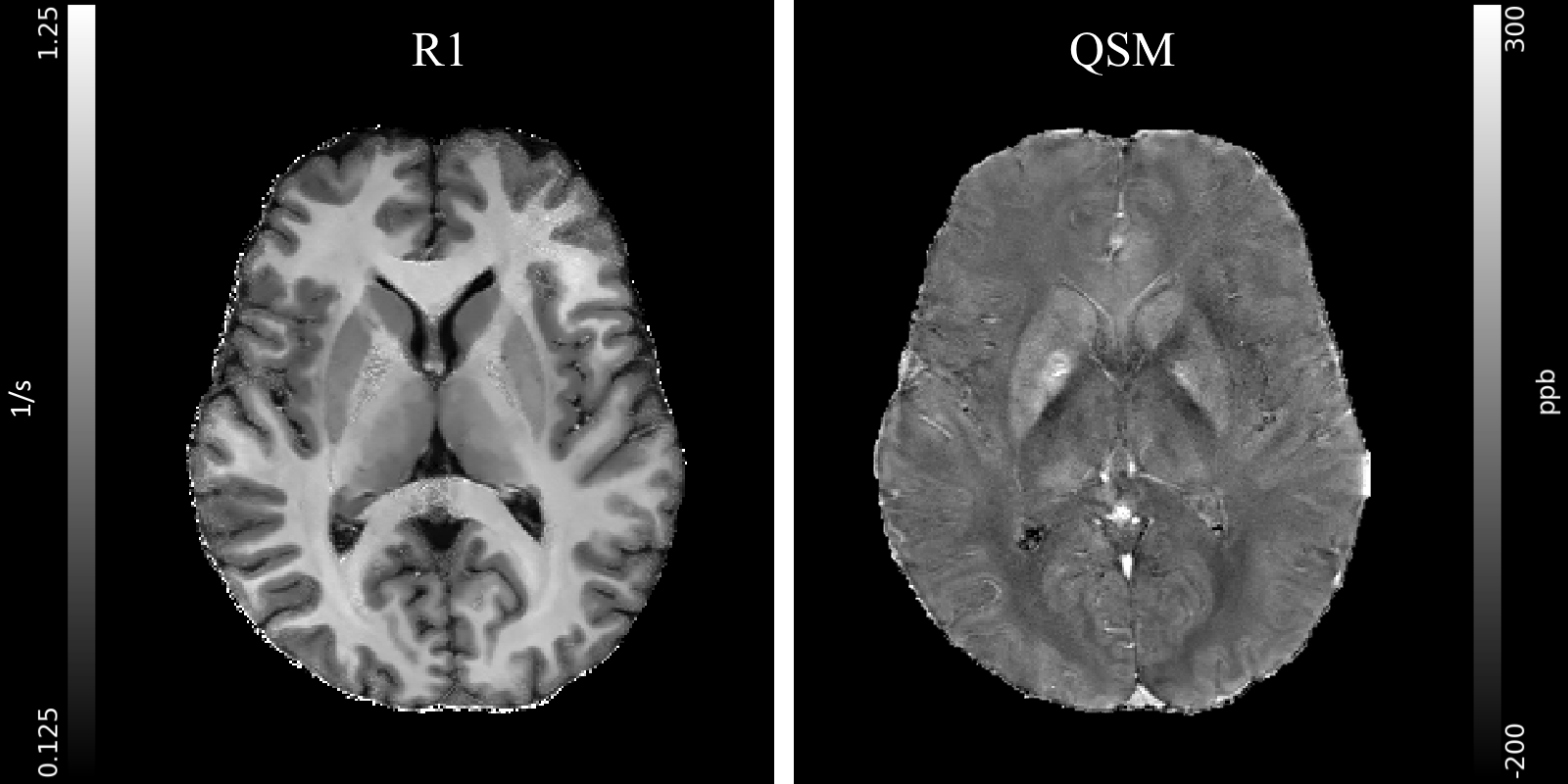

Supplement: Supplementary file 1 — (PNG 115 kb) [file 330_2022_9154_Fig6_ESM.png]

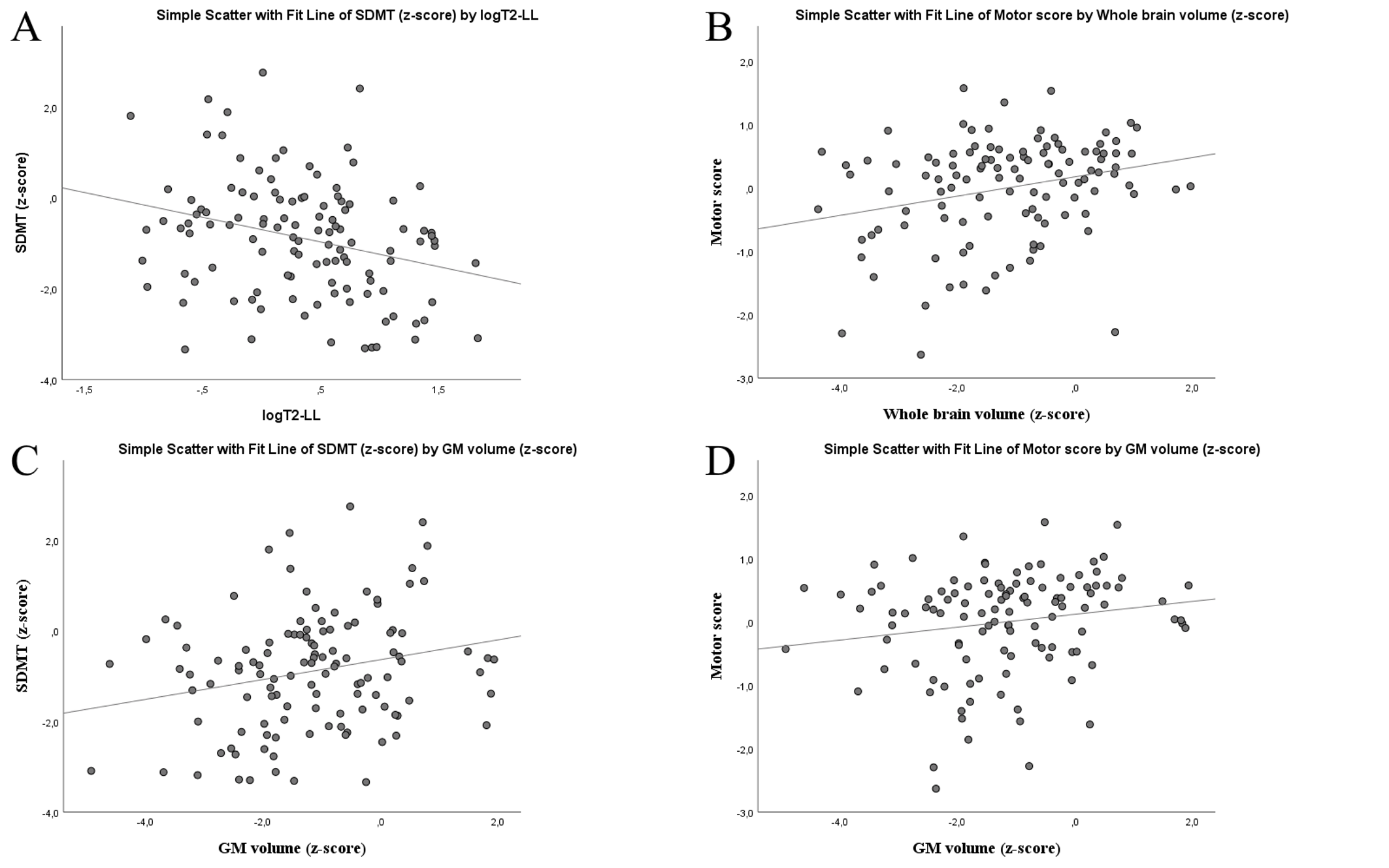

Supplement: Supplementary file 3 — (PNG 333 kb) [file 330_2022_9154_Fig7_ESM.png]

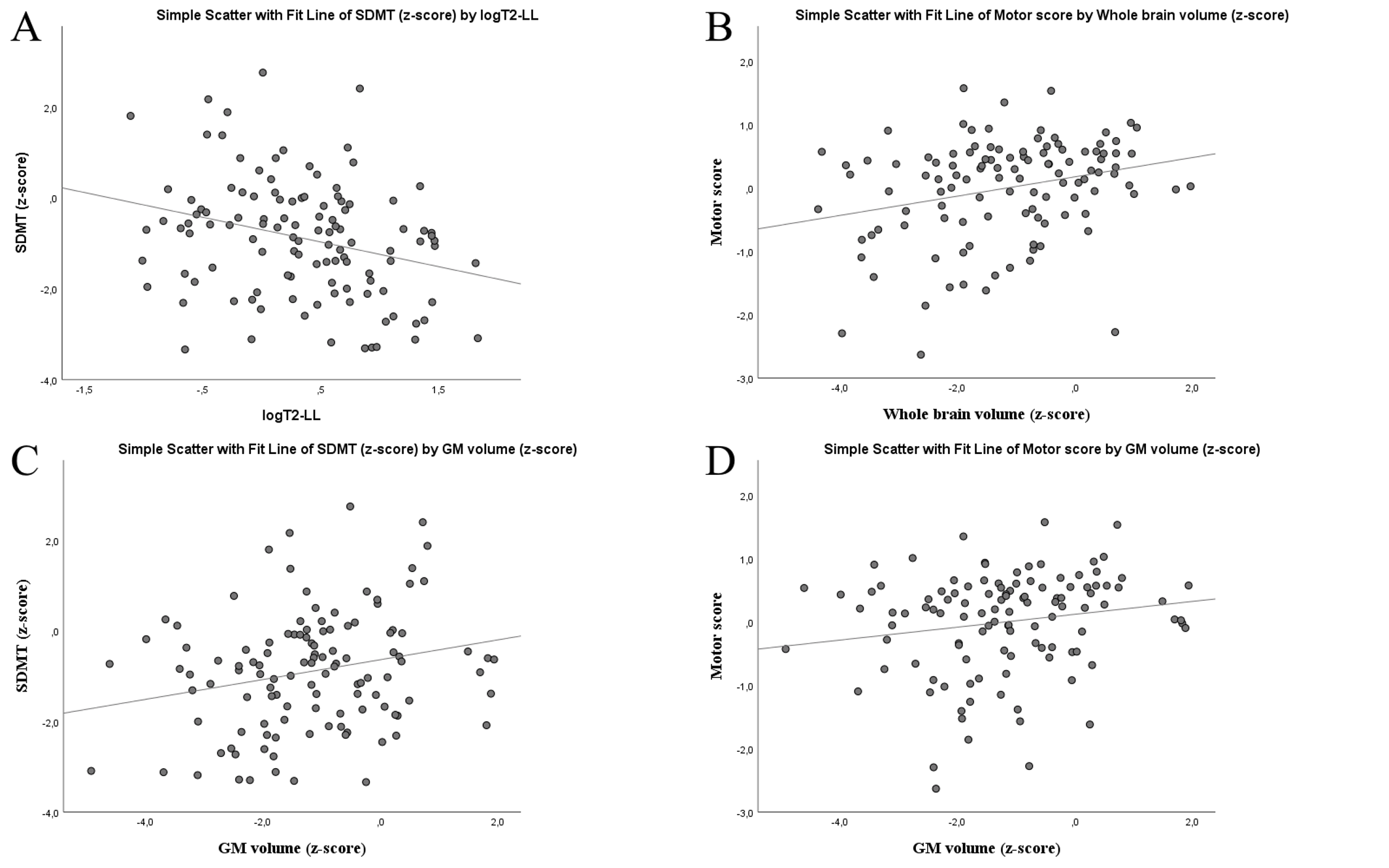

Supplement: Supplementary file 4 — High Resolution Image (TIFF 512 kb) [file 330_2022_9154_MOESM2_ESM.tif]
