# Supplementary material for: Clinical correlates of R1 relaxometry and magnetic susceptibility changes in multiple sclerosis: a multi-parameter quantitative MRI study of brain iron and myelin
Source: Eur Radiol. 2022 Oct 14;33(3):2185–94. doi: 10.1007/s00330-022-09154-y (PMC9935712; doi:10.1007/s00330-022-09154-y)
Supplement: Supplementary file 5 — (DOCX 55.2 kb) [file 330_2022_9154_MOESM3_ESM.docx]

**Clinical correlates of R1 relaxometry and magnetic susceptibility changes in Multiple Sclerosis: a multi-parameter quantitative MRI study of brain iron and myelin**

**Methods**

*MRI protocol*

All MRI exams were performed on the same 3T scanner (Magnetom Trio, Siemens Healthineers), equipped with an 8-channel head coil, with the following protocol: a 3D T1-weighted Magnetization Prepared Rapid Acquisition Gradient Echo sequence (MPRAGE; TR=2500 ms; TE=2.8 ms; TI=900 ms; Flip Angle, FA=9°; voxel size=1x1x1 mm^3^; 160 axial slices) for volumetric analyses; a 3D T2-weighted Fluid Attenuated Inversion Recovery sequence (FLAIR; TR=6000 ms; TE=396 ms; TI=2200 ms; FA=120°; voxel size=1x1x1 mm^3^; 160 sagittal slices) for T2-hyperintense lesions detection and lesion load (T2-LL) quantification; a single-echo spoiled gradient echo sequence (TR=16 ms; TE=7.38 ms; FA=2°) and a dual-echo flow-compensated spoiled gradient echo sequence (TR=32 ms; TE1=7.38 ms and TE2=22.14 ms; FA=20°), with the same geometry (voxel size=0.5×0.5×1 mm^3^; 160 axial slices) for quantitative analyses.

*R1 and QSM maps computation*

A complete description of the processing steps leading to the computation of longitudinal relaxation rate (R1) and QSM (χ) maps is available in previous works[1-3]. Briefly, the R1 map is obtained by a variable FA scheme, optimized in order to reduce the acquisition time of the low FA sequence by halving the TR. The typical biases introduced by non-ideal excitation and RF-spoiling are corrected in post-processing by minimizing the entropy of the R1 distribution as a function of a second-order polynomial FA map and according to the method described in[4]. On the other hand, the QSM is derived from the dual-echo GRE following the approach described in[5], using the STI Suite software (<https://people.eecs.berkeley.edu/~chunlei.liu/software.html)>. Representative R1 and quantitative susceptibility maps are shown in Supplementary Figure 1.

*Spatial preprocessing*

Initially, to take into account possible differences in terms of spatial orientation, T1-weighted and FLAIR images were automatically reoriented by rigidly aligning them to corresponding templates in the MNI space using the Statistical Parametric Mapping software package (SPM12, http://www.fil.ion.ucl.ac.uk/spm).

Quantitative maps were then mapped onto the corresponding T1-weighted volumes through affine co-registration of R1 maps using normalized cross-correlation as similarity metric. Demyelinating lesions were automatically segmented on FLAIR images using the lesion prediction algorithm implemented in the Lesion Segmentation Tool (LST) toolbox v3.0.0 (www.statistical-modelling.de/lst.html) for SPM. Individual lesion probability maps where then used to fill lesions in T1-weighted images for subsequent processing steps via LST’s default lesion filling procedure, and binarized (thresholding at 0.5 probability) to compute T2-LL.

Subsequent spatial processing steps were carried out following the voxel-based morphometry (VBM)[6] and voxel-based quantification (VBQ)[7] approaches. Briefly, filled T1-weighted volumes were segmented into different tissue classes and normalized to a 1mm isotropic template in MNI space via the standard pipeline implemented in the Computational Anatomy Toolbox (CAT12.7, http://www.neuro.uni-jena.de/cat) for SPM, with extended tissue priors to ensure better classification of subcortical GM structures[8]. The estimated spatial transformations were then applied to quantitative maps to bring them in alignment with the common space. Finally, as in standard VBM preprocessing[6], normalized GM and WM probability maps were modulated with the Jacobian determinant derived from the spatial normalization and smoothed using a 1mm full width at half maximum (FWHM) isotropic Gaussian kernel[9]. On the other hand, for VBQ, normalized R1 and χ maps were smoothed (1-mm FWHM isotropic Gaussian kernel) while accounting for the partial volume contribution of the tissue density in each voxel[7] via the tissue-weighted smoothing procedure implemented in the hMRI toolbox (https://hmri-group.github.io/hMRI-toolbox)[10] for SPM, resulting in tissue-specific (i.e. GM and WM) smoothed quantitative maps in MNI space.

For each participant, total intracranial volume (TIV) was estimated using CAT12 standard procedure and brain parenchymal, GM and WM fractions (BPf, GMf, WMf) were computed as volume ratios to TIV. Additionally, normal-appearing GM (NAGM) and WM (NAWM) masks were obtained by subtracting the lesion mask from the corresponding tissue mask and used to extract median values of R1 and χ. Moreover, to further reduce possible spurious atrophy-related effects on VBQ analyses, as well as to ensure that each voxel was analyzed in only one subspace (i.e. GM or WM), explicit GM and WM masks were obtained and used to restrict voxel-wise statistical analyses [11]: normalized, modulated and smoothed individual GM, WM and CSF maps were averaged across all subjects, thresholded at 20%, and corresponding masks were obtained assigning each voxel the tissue class for which the probability was maximal.

**Supplementary Tables.**

**Supplementary Table 1. Clusters of significant regional volume, R1 and** χ **changes in MS patients compared to HC for both the MS > HC and MS < HC contrasts**. Each cluster’s volume is presented, along with the corresponding local maxima’s significance level (FWE-corrected), TFCE statistics, effect sizes and locations. Coordinates refer to mm from the anterior commissure in MNI space, with anatomical labeling according to the AAL and Mori atlases implemented in CAT12 for GM and WM regions, respectively.

| **Contrast** | | **Cluster Volume (ml)** | ***p*-value**  **(FWE-corrected)** | **TFCE** | **Cohen’s *d**** | **MNI coordinates (mm)** | | | **Anatomical Label** |
| --- | --- | --- | --- | --- | --- | --- | --- | --- | --- |
|  |  |  |  |  |  | **X** | **Y** | **Z** |  |
| **Volume** | |  |  |  |  |  |  |  |  |
| GM | MS < HC | 300.6 | <0.001 | 15657.05 | 1.30 | -14.5 | -30.5 | 2.5 | Left Thalamus |
|  |  |  | <0.001 | 15641.30 | 1.29 | 1.5 | -15.5 | 4.5 | Right Thalamus |
|  |  | 6.7 | 0.004 | 1986.49 | 0.83 | -30.5 | -67.5 | -39.5 | Left Cerebellar Crus II |
|  |  |  | 0.004 | 1930.58 | 0.80 | -24.5 | -73.5 | -35.5 | Left Cerebellar Crus I |
|  |  | 0.3 | 0.014 | 570.79 | 0.46 | 17.5 | -85.5 | 32.5 | Right Superior Occipital Gyrus |
|  |  |  | 0.014 | 561.25 | 0.42 | 10.5 | -84.5 | 36.5 | Right Cuneus |
| WM | MS < HC | 254.2 | <0.001 | 7544.47 | 1.23 | 22.5 | -32.5 | 7.5 | Right Fornix Stria Terminalis |
| **R1** | |  |  |  |  |  |  |  |  |
| GM | MS < HC | 381.4 | <0.001 | 12467.08 | 1.61 | -21.5 | -27.5 | -6.5 | Left Thalamus |
|  |  |  | <0.001 | 11919.51 | 1.39 | 20.5 | -27.5 | -4.5 | Right Thalamus |
|  |  | 0.1 | 0.015 | 2288.78 | 0.34 | -12.5 | -44.5 | 70.5 | Left Precuneus |
|  |  |  | 0.038 | 1415.78 | 0.47 | -16.5 | -37.5 | 76.5 | Left Postcentral Gyrus |
|  |  | 0.2 | 0.030 | 1612.56 | 0.50 | -2.5 | -79.5 | 14.5 | Left Cuneus |
|  |  |  | 0.031 | 1597.12 | 0.52 | 6.5 | -75.5 | 22.5 | Right Cuneus |
|  |  | 0.5 | 0.035 | 1489.32 | 0.86 | 24.5 | 52.5 | 9.5 | Right Superior Frontal Gyrus |
|  |  |  | 0.045 | 1272.99 | 0.55 | 29.5 | 54.5 | 3.5 | Right Middle Frontal Gyrus |
|  |  | 0.7 | 0.035 | 1485.15 | 0.68 | -35.5 | 45.5 | 23.5 | Left Middle Frontal Gyrus |
|  |  |  | 0.036 | 1452.62 | 0.58 | -43.5 | 41.5 | 0.5 | Left Inferior Frontal Gyrus |
|  |  | 0.2 | 0.036 | 1467.37 | 0.87 | -39.5 | -17.5 | 45.5 | Left Postcentral Gyrus |
|  |  | 0.2 | 0.038 | 1408.96 | 0.54 | 22.5 | -58.5 | 57.5 | Right Superior Parietal Gyrus |
|  |  | 0.2 | 0.038 | 1408.07 | 0.53 | -27.5 | -92.5 | 6.5 | Left Middle Occipital Gyrus |
|  |  | 0.2 | 0.039 | 1396.24 | 0.91 | -34.5 | -27.5 | 62.5 | Left Postcentral Gyrus |
|  |  |  | 0.044 | 1286.42 | 0.65 | -27.5 | -26.5 | 55.5 | Left Precentral Gyrus |
|  |  | 0.2 | 0.042 | 1328.73 | 0.66 | -27.5 | -40.5 | 62.5 | Left Superior Parietal Gyrus |
|  |  |  | 0.049 | 1199.58 | 0.38 | -19.5 | -39.5 | 63.5 | Left Postcentral Gyrus |
|  |  | 0.2 | 0.042 | 1325.79 | 0.77 | -18.5 | -30.5 | 61.5 | Left Postcentral Gyrus |
|  |  | 0.2 | 0.045 | 1268.70 | 0.43 | 10.5 | 15.5 | 8.5 | Right Superior Frontal Gyrus |
|  |  | 0.1 | 0.047 | 1240.63 | 0.56 | 57.5 | -13.5 | 39.5 | Right Postcentral Gyrus |
| WM | MS < HC | 543.1 | <0.001 | 31878.48 | 2.35 | 0.5 | 20.5 | 8.5 | Left Corpus Callosum (Genu) |
|  |  |  | <0.001 | 29928.05 | 1.86 | 1.5 | 13.5 | 16.5 | Left Corpus Callosum (Body) |
| **χ** | |  |  |  |  |  |  |  |  |
| GM | MS < HC | 1.6 | <0.001 | 1729.52 | 1.05 | 16.5 | -32.5 | 3.5 | Right Thalamus |
|  |  | 1.1 | <0.001 | 1330.70 | 0.85 | -13.5 | -31.5 | 9.5 | Left Thalamus |
|  |  | 0.1 | 0.014 | 865.33 | 0.78 | 19.5 | -31.5 | -7.5 | Right Hippocampus |
|  | MS > HC | 0.7 | 0.005 | 1012.04 | -0.84 | 4.5 | 36.5 | 23.5 | Right Anterior Cingulate Gyrus |
|  |  |  | 0.010 | 939.14 | -0.72 | 6.5 | 29.5 | 29.5 | Right Middle Cingulate Gyrus |
|  |  | 0.3 | 0.016 | 876.50 | -0.75 | 22.5 | 37.5 | 42.5 | Right Superior Frontal Gyrus |
|  |  | 0.1 | 0.004 | 1382.12 | -1.14 | -19.5 | -17.5 | 26.5 | Left Caudate |
| WM | MS < HC | 34.5 | <0.001 | 2739.79 | 1.02 | -15.5 | 21.5 | 27.5 | Left Anterior Corona Radiata |
|  |  |  | <0.001 | 2113.08 | 0.79 | -32.5 | -21.5 | 35.5 | Left Superior Longitudinal Fasciculus |
|  |  | 17.8 | <0.001 | 2412.08 | 0.96 | 14.5 | -14.5 | 31.5 | Right Corpus Callosum (Body) |
|  |  |  | <0.001 | 2372.83 | 0.88 | 17.5 | -9.5 | 38.5 | Right Superior Corona Radiata |
|  |  | 1.2 | 0.001 | 1501.15 | 0.98 | 0.5 | -24.5 | -5.5 | Left Midbrain |
|  |  |  | 0.011 | 1167.71 | 0.67 | 6.5 | -23.5 | -13.5 | Right Midbrain |
|  |  | 3.0 | 0.009 | 1199.43 | 0.75 | 49.5 | -17.5 | 34.5 | Right Postcentral Gyrus |
|  |  |  | 0.014 | 1113.13 | 0.66 | 33.5 | -21.5 | 39.5 | Right Superior Longitudinal Fasciculus |
|  |  | 3.5 | 0.010 | 1184.61 | 0.65 | 15.5 | 54.5 | 8.5 | Right Superior Frontal Gyrus |
|  |  | 0.8 | 0.016 | 1093.55 | 0.67 | 48.5 | -31.5 | 11.5 | Right Superior Temporal Gyrus |
|  |  | 0.5 | 0.020 | 1061.59 | 0.68 | -55.5 | -17.5 | -0.5 | Left Superior Temporal Gyrus |
|  |  | 0.2 | 0.025 | 1017.42 | 0.64 | -35.5 | -14.5 | 11.5 | Left Insula |
|  |  | 0.3 | 0.030 | 986.26 | 0.53 | 46.5 | -12.5 | 22.5 | Right Postcentral Gyrus |
|  |  | 0.2 | 0.037 | 951.65 | 0.56 | 32.5 | -53.5 | 38.5 | Right Angular Gyrus |
|  |  | 0.1 | 0.042 | 927.64 | 0.50 | 50.5 | -43.5 | 27.5 | Right Angular Gyrus |

* Estimated from the non-parametric T statistic obtained with the TFCE toolbox and referred to the MS < HC contrast.

HC=healthy controls; FWE=family-wise error; TFCE=Threshold-Free Cluster Enhancement; MNI=Montreal Neurological Institute; CAT=Computational Anatomy Toolbox; AAL=Automated Anatomical Labeling.

**Supplementary Table 2. Clusters of significant association between regional volume, R1 and** ꭓ **changes and clinical variables (i.e. EDSS, SDMT and motor scores) in MS patients**. Each cluster’s volume is presented, along with the corresponding local maxima’s significance level (FWE-corrected), TFCE statistics, effect sizes and locations. Coordinates refer to mm from the anterior commissure in MNI space, with anatomical labeling according to the AAL and Mori atlases implemented in CAT12 for GM and WM regions, respectively.

| **Contrast** | **Cluster Volume (ml)** | ***p*-value**  **(FWE-corrected)** | **TFCE** | **Correlation Coefficient**  **(*r*)***** | **MNI coordinates (mm)** | | | **Anatomical Label** |
| --- | --- | --- | --- | --- | --- | --- | --- | --- |
|  |  |  |  |  | **X** | **Y** | **Z** |  |
| **EDSS** |  |  |  |  |  |  |  |  |
| Volume (GM) | 9.5 | 0.001 | 1493.65 | -0.45 | -3.5 | -5.5 | 5.5 | Left Thalamus |
| χ (WM) | 3.4 | <0.001 | 923.36 | -0.39 | -10.5 | 4.5 | 45.5 | Left Cingulate Gyrus |
|  |  | 0.001 | 844.79 | -0.35 | -10.5 | 6.5 | 53.5 | Left Superior Frontal Gyrus |
|  | 0.6 | 0.005 | 637.32 | -0.40 | 7.5 | -17.5 | 32.5 | Right Cingulum and Cingulate Gyrus |
|  |  | 0.008 | 583.69 | -0.35 | 9.5 | -10.5 | 37.5 | Right Cingulate Gyrus |
| **SDMT** |  |  |  |  |  |  |  |  |
| Volume (GM) | 11.0 | 0.001 | 1430.18 | 0.38 | 12.5 | -15.5 | 15.5 | Right Thalamus |
|  |  | 0.001 | 1420.28 | 0.41 | -16.5 | -21.5 | 7.5 | Left Thalamus |
|  | 3.6 | 0.009 | 942.75 | 0.40 | 28.5 | -3.5 | -5.5 | Right Pallidum |
|  |  | 0.011 | 902.35 | 0.42 | 32.5 | 4.5 | -1.5 | Right Putamen |
|  | 0.7 | 0.039 | 626.33 | 0.32 | 19.5 | 17.5 | 3.5 | Right Caudate Nucleus |
| R1 (WM) | 55.1 | 0.011 | 2944.12 | 0.42 | 42.5 | -39.5 | 4.5 | Right Middle Temporal Gyrus |
|  |  | 0.011 | 2928.77 | 0.41 | 39.5 | -42.5 | -2.5 | Right Posterior Thalamic Radiation |
|  |  | 0.012 | 2853.32 | 0.38 | 27.5 | -64.5 | 1.5 | Right Lingual Gyrus |
| **Motor score** |  |  |  |  |  |  |  |  |
| Volume (GM) | 5.3 | 0.014 | 914.08 | 0.36 | -1.5 | -6.5 | 8.5 | Left Thalamus |
| Volume (WM) | 3.8 | 0.010 | 2151.79 | 0.50 | 9.5 | -37.5 | -42.5 | Right Medial Lemniscus /  Right Cerebellar Peduncles |
|  |  | 0.014 | 1958.30 | 0.51 | -8.5 | -40.5 | -44.5 | Left Cerebellar Peduncles |
| χ (WM) | 0.8 | 0.003 | 808.41 | 0.45 | 10.5 | -16.5 | 33.5 | Right Cingulum and Cingulate Gyrus |
|  |  | 0.012 | 597.05 | 0.37 | 18.5 | -14.5 | 40.5 | Right Superior Corona Radiata |
|  | 0.6 | 0.019 | 537.44 | 0.37 | -23.5 | -6.5 | 40.5 | Left Superior Frontal Gyrus |
|  |  | 0.021 | 527.99 | 0.35 | -15.5 | -3.5 | 40.5 | Left Superior Corona Radiata |

* Estimated from the non-parametric T statistic obtained with the TFCE toolbox and referred to the positive association contrast.

HC=healthy controls; FWE=family-wise error; TFCE=Threshold-Free Cluster Enhancement; MNI=Montreal Neurological Institute; EDSS=Expanded Disability Status Scale; SDMT=Symbol Digit Modalities Test; CAT=Computational Anatomy Toolbox

**Supplementary Figures**

**Supplementary Figure 1. R1 and quantitative susceptibility maps.** Representative axial slices of R1 and quantitative susceptibility maps of a 45-year-old male healthy subject.

**Supplementary Figure 2. Scatter plots showing the relationship between global MRI metrics and clinical scores for significant correlations.** Scatter plots showing the relationships between log-transformed T2-LL and SDMT (A), age-, sex- and TIV-adjusted z-scores of whole brain volume and motor score (B) and age-, sex- and TIV-adjusted z-scores of GM volume with SDMT (C) and motor (D) scores, respectively. Effect sizes (with 95% confidence intervals) and statistical significance values are reported in Table 2.

**References**

1 Palma G, Tedeschi E, Borrelli P et al (2015) A Novel Multiparametric Approach to 3D Quantitative MRI of the Brain. PLoS One 10:e0134963

2 Borrelli P, Palma G, Tedeschi E et al (2015) Improving Signal-to-Noise Ratio in Susceptibility Weighted Imaging: A Novel Multicomponent Non-Local Approach. PLoS One 10:e0126835

3 Monti S, Borrelli P, Tedeschi E, Cocozza S, Palma G (2017) RESUME: Turning an SWI acquisition into a fast qMRI protocol. PLoS One 12:e0189933

4 Baudrexel S, Nöth U, Schüre J-R, Deichmann R (2018) T1 mapping with the variable flip angle technique: A simple correction for insufficient spoiling of transverse magnetization. Magn Reson Med 79:3082-3092

5 Liu C, Li W, Tong KA, Yeom KW, Kuzminski S (2015) Susceptibility-weighted imaging and quantitative susceptibility mapping in the brain. J Magn Reson Imaging 42:23-41

6 Ashburner J, Friston KJ (2000) Voxel-based morphometry--the methods. Neuroimage 11:805-821

7 Draganski B, Ashburner J, Hutton C et al (2011) Regional specificity of MRI contrast parameter changes in normal ageing revealed by voxel-based quantification (VBQ). Neuroimage 55:1423-1434

8 Lorio S, Fresard S, Adaszewski S et al (2016) New tissue priors for improved automated classification of subcortical brain structures on MRI. Neuroimage 130:157-166

9 Smith SM, Nichols TE (2009) Threshold-free cluster enhancement: addressing problems of smoothing, threshold dependence and localisation in cluster inference. Neuroimage 44:83-98

10 Tabelow K, Balteau E, Ashburner J et al (2019) hMRI - A toolbox for quantitative MRI in neuroscience and clinical research. Neuroimage 194:191-210

11 Callaghan MF, Freund P, Draganski B et al (2014) Widespread age-related differences in the human brain microstructure revealed by quantitative magnetic resonance imaging. Neurobiol Aging 35:1862-1872
